# Draft Genome Sequence of *Asaia* sp. Strain SF2.1, an Important Member of the Microbiome of *Anopheles* Mosquitoes

**DOI:** 10.1128/genomeA.01202-13

**Published:** 2014-01-09

**Authors:** Jackie L. Shane, Nicholas J. Bongio, Guido Favia, David J. Lampe

**Affiliations:** Department of Biological Sciences, Duquesne University, Pittsburgh, Pennsylvania, USAa; Scuola di Bioscienze e Biotecnologie, Università degli Studi di Camerino, Camerino, Italyb

## Abstract

*Asaia* spp. are abundant members of the microbiota of *Anopheles* mosquitoes, the principle vectors of malaria. Here, we report the draft genome sequence of *Asaia* sp. strain SF2.1. This strain is under development as a platform to deliver antimalarial peptides and proteins to adult female *Anopheles* mosquitoes.

## GENOME ANNOUNCEMENT

*Asaia* sp. strain SF2.1 is a Gram-negative member of the *Alphaproteobacteria*, family *Acetobacteraceae* (1). *Asaia* spp. were first isolated from the nectar of tropical flowers and subsequently from insect midguts (1–4). The taxonomy of the genus *Asaia* is in flux and so we are hesitant to assign *Asaia* sp. SF2.1 to a specific species, although it seems to be most closely related to either *Asaia bogorensis* or *Asaia platycodi* (our unpublished data). *Asaia* sp. SF2.1 was isolated from a laboratory colony of *Anopheles stephensi,* where it is extremely abundant in the gut, salivary glands, ovaries, and testes of this insect (5). *Asaia* spp. have also been uncovered in *Anopheles* mosquitoes in the field, especially *Anopheles gambiae*, the most important vector of malaria in Africa (2, 6, 7). Efforts to genetically engineer *Asaia* sp. SF2.1 are under way in order to provide a platform to deliver antimalarial effector molecules to *Anopheles* mosquitoes in the field in an effort to block the transmission of malaria, a strategy called paratransgenesis (7–9). The sequence reported here is the first for the genus.

The sequencing and annotation of the genome of *Asaia* sp. SF2.1 was performed by ACGT, Inc. The standard protocol for the Nextera XT DNA sample preparation kit was used. The purified fragmented DNA was used as a template for a limited cycle PCR using Nextera primers and index adaptors. A second library was prepared using the Nextera mate-pair sample preparation kit.

In order to generate clusters of DNA, both libraries were sequenced in a paired-end 2 × 150-bp protocol by MiSeq. The sequence reads passing the Illumina purity filter were demultiplexed. A total of 4,097,892 standard library reads were generated, giving an average coverage of 351× based on the 3.5-Mb genome. To generate additional mate-pair reads, a second MiSeq run was done using the mate-pair library. This run generated 999,241 mate-pair reads (86× coverage).

A 93× coverage subset of the small-insert library and the mate-pair library were assembled *de novo* using ABySS (10), Velvet (11), and SOAP*denovo*2 (12). The best Velvet, ABySS, and SOAP*denovo*2 contig sets were combined using CISA (13) to produce an assembly with 51 contigs. The largest contig is 506 kb, the N_50_ length is 162 kb, and the total assembly length is 3.53 Mb. The G+C content is 59.5%.

Annotation of the genome was performed by the NCBI Prokaryotic Genome Annotation Pipeline version 2.0 (https://www.ncbi.nlm.nih.gov/genome/annotation_prok/). A total of 3,098 genes were predicted using this method, including 3,005 protein-coding genes, 44 pseudogenes, 3 rRNAs, and 45 tRNAs.

### Nucleotide sequence accession numbers.

This whole-genome shotgun project has been deposited at DDBJ/EMBL/GenBank under the accession no. AYXS00000000. The version described in this paper is version AYXS01000000.
